# Gene expression analysis of matched ovarian primary tumors and peritoneal metastasis

**DOI:** 10.1186/1479-5876-10-121

**Published:** 2012-06-11

**Authors:** Joel A Malek, Alejandra Martinez, Eliane Mery, Gwenael Ferron, Ruby Huang, Christophe Raynaud, Eva Jouve, Jean-Paul Thiery, Denis Querleu, Arash Rafii

**Affiliations:** 1 Genomics Core, Weill Cornell Medical College in Qatar, Education city, Qatar Foundation, Doha, Qatar, PO 241 44; 2 Department of surgical Oncology, and Pathology, Institut Claudius Regaud, Toulouse, F-31052, France; 3 Department of Obstetrics and Gynecology and Cancer Science institute of Singapore, National University of Singapore, Singapore, 117456, Singapore; 4 Stem cell and microenvironment laboratory, Weill Cornell Medical College in Qatar, Education city, Qatar Foundation, Doha, Qatar, PO 24144; 5 Institute of Molecular and Cell Biology, A*STAR, Singapore, 138673, Singapore; 6 Department of Obstetrics and Gynecology, McGill University, Montreal, Canada; 7 Department of Genetic Medicine, Weill Cornell Medical College, NY, NY, USA

## Abstract

**Background:**

Ovarian cancer is the most deadly gynecological cancer due to late diagnosis at advanced stage with major peritoneal involvement. To date most research has focused on primary tumor. However the prognosis is directly related to residual disease at the end of the treatment. Therefore it is mandatory to focus and study the biology of meatastatic disease that is most frequently localized to the peritoneal caivty in ovarian cancer.

**Methods:**

We used high-density gene expression arrays to investigate gene expression changes between matched primary and metastatic (peritoneal) lesions.

**Results:**

Here we show that gene expression profiles in peritoneal metastasis are significantly different than their matched primary tumor and these changes are affected by underlying copy number variation differences among other causes. We show that differentially expressed genes are enriched in specific pathways including JAK/STAT pathway, cytokine signaling and other immune related pathways. We show that underlying copy number variations significantly affect gene expression. Indeed patients with important differences in copy number variation displayed greater gene expression differences between their primary and matched metastatic lesions.

**Conclusions:**

Our analysis shows a very specific targeting at both the genomic and transcriptomic level to upregulate certain pathways in the peritoneal metastasis of ovarian cancer. Moreover, while primary tumors use certain pathways we identify distinct differences with metastatic lesions. The variation between primary and metastatic lesions should be considered in personalized treatment of ovarian cancer.

## Background

Epithelial Ovarian carcinoma (EOC) is the sixth most common malignancy in women and the leading cause of death from gynecological cancer [1]. The poor overall survival (20 to 30% at 5 years) is due to large tumor burden with extensive peritoneal metastatic lesions. Abdominal recurrences remain an issue and result in patients’ poor prognosis despite ultra-radical surgical procedures and initial chemosensitivity. Advanced ovarian cancer therefore represents a unique situation with most patients having progressed to the metastatic stage but the treatment still aims at cure. The high rate of peritoneal recurrences after debulking surgery and chemotherapy indicates our failure to correctly target the metastatic lesions. Indeed it has been demonstrated that peritoneal residual disease (even below 5 mm) impacts prognosis [2]. This has led to a change of practice with a combination of ultra-radical surgeries and chemotherapy to achieve complete (no macroscopic) tumor residue.

Therefore with the advent of personalized medicine it is critical to understand the molecular pathways underlying peritoneal metastasis in order to be able to define new therapeutic strategies. This will require understanding the genetic variation in both primary and metastatic lesions to correctly optimize the therapy on an individual basis. One method to distinguish these differences is the use of high-density gene expression arrays that will allow identifying genes specifically implicated in pathological processes. Much focus has been placed on identifying gene expression differences between normal ovarian tissue and ovarian cancer to understand the drivers of carcinogenesis [3,4]. Several studies have also tried to delineate gene expression signatures for prognostic predictions as well as chemotherapeutic responses [5-7]. These studies have attempted to provide gene predictors on disease outcome, however, the robustness and reproducibility of these genes lists across different patient populations have not yet been clearly established or translated to clinical practice [8]. The question remains, however, what the genetic requirements are on the primary tumor cells involved in the progression to metastasis. Lancaster et al. have compared primary ovarian tumor and omental metastatic lesions in a study of 20 patients. They found Fifty-six genes with differential expression between primary ovarian tumors and omental metastatic samples. The genes uncovered by their approach were previously implicated in the metastatic process, cell motility, migration, and cytoskeletal function. Critical networks such as the p53 pathway were also enriched [9]***.***

We recently investigated copy number variations (CNVs) between matched primary and metastatic ovarian tumors [10] and identified large scale differences between the primary lesions and metastatic lesions and suggested those changes would likely affect gene expression as well.

We further hypothesized that gene expression variability may be due to the targeting of genomic amplification or deletion of specific pathways rather than specific genes themselves. This would result in gene expression changes that averaged to insignificance when observed across multiple individuals. However, if gene expression is considered in the light of copy number variations, affected pathways relevant to specific patients will more likely to be identified.

In this follow up study to our analysis of CNVs we demonstrate transcriptomic differences with potential therapeutic implication between primary and metastatic lesions.

## Methods

### Ethics statement and sample collection/preparation

All the samples were collected in the department of Gynecologic Oncology at the Institut Claudius Regaud (DQ, AR, AM, GF). The project was reviewed and approved by the institution’s Human research Ethics Committee. All patients included in the study gave informed written consent prior to surgery. 9 patients with advance Stage III or IV papillary serous ovarian adenocarcinoma were prospectively enrolled in this study at the time of primary surgery before any treatment was given (Table 1). The patients had a biopsy of the primary lesion as well as a peritoneal metastasis outside of the pelvis. In order to ensure very little contamination by the stromal components the biopsies specifically took the tumoral nodules without the underlying peritoneal elements. All biopsies were immediately liquid nitrogen snap frozen. A representative haematoxylin and eosin stained section from the snap frozen was assessed and samples with 80% epithelial cells and less than 20% of necrosis (criteria used by the TCGA group [11]) were used for DNA and RNA extraction from the whole tissue.

**Table 1:** Patient Information used in this study

**Age**	**61 +/− 7**
Histology (9 patients)	Papillary-serous adenocarcinoma
Grade (9 patients)	3
StageIIICIV (pleural)	8 1 (OV07-5)
Adjuvant treatment	Carboplatin and taxol (6 cycles)

### RNA and DNA isolation

DNA and RNA were isolated using QIA-cube technology as per the manufacturer instructions.

### Gene expression arrays

We used the Affymetrix Human Gene 1.0 chip for detection of gene expression levels in this study. The manufacturers protocol for the Affymetrix Human Gene 1.0 chip was strictly followed. 100 ng of total RNA were used in the analysis.

### Data analysis

#### Gene expression analysis

**Figure 1:**
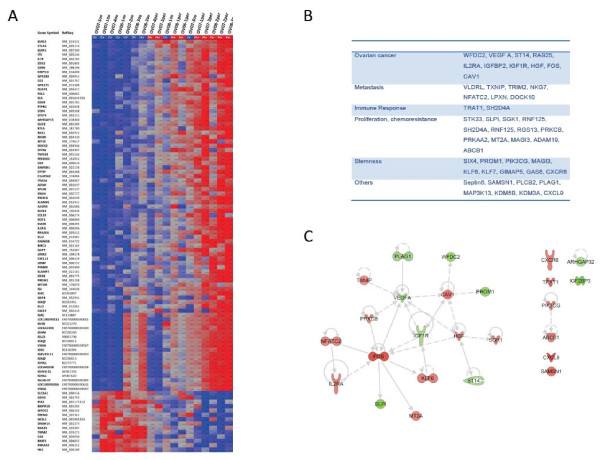
**A. Clustering of Tumors based on genes differentially expressed between ovarian primary tumors and their matched peritoneal metastasis.** A fold change cut-off of 2 was used. Normalized Signal intensity levels are plotted for each tumor type at genes differentially expressed between the tumor types. The Primary tumor was used to create a baseline average. Patient IDs and tumor type are displayed above the plot. Red: overexpression, Blue:Underexpressed. Ov: ovarian tumor, Pe: peritoneal metastasis. **B**. List of most relevant genes associated to neoplasic disease. **C.** A network representing all genes implicated in the “cancer” category is represented in Figure 1C and demonstrates the central role of IGF, VEGF, CAV1 and FOS.

Data from the Affymetrix Human Gene 1.0 arrays were analyzed using the PARTEK Genomics Suite software with recommended normalization (RMA) settings. Comparison of gene expression levels between primary tumor and peritoneal metastasis was conducted using the paired *T*-test. Primary ovarian tumors were used as the baseline in this study. Only genes with at least 1.5 fold change and paired *T*-test p-value of less than 0.01 were reported (Additional file 1: Table 1). A subset with fold change of 2 is shown in Figure 1. We opted to use a relatively low cut-off for fold change (1.5 versus the typical 2 to 3 fold) based on observations from the CNV data. Specifically, a single allele gain in primary or metastatic tumors would result in a predicted 1.5 fold change in gene expression assuming all other factors remained constant. Additionally, our goal was to use lower significance cutoffs yet follow up with more stringent pathway enrichment analysis. This approach has been shown to yield significant functional significance despite initial lack of strong statistical significance [12]. Gene lists from gene expression were entered into DAVID [13] and KEGG pathways enriched with Benjamini-Hochberg score of less than 0.25 were selected.

### Overlap of gene expression with copy number variations

Genomics coordinates of Copy Number Variations in the genomic DNA of matched samples from this study were obtained [10]. Coordinates of probe sets from the Affymetrix Gene 1.0 chip were mapped to CNVs. Only those probe sets, representing the whole gene transcript and which were fully encompassed by a CNV segment were analyzed. Probe set intensities from the gene expression analysis for each individual (see above) were then separated into groups based on amplified, deleted or normal in a given CNV segment in the respective patient. Gene expression data for each patient at each gene was matched to CNV locations. Gene expression data was then divided into categories at each gene depending on whether the patient was amplified, deleted or unaffected in that gene (see Methods). Gene expression analysis was conducted as CNV segments and a segment required at least 3 patients’ data within it. For example, all patients showing amplification in a gene had their matched peritoneum to primary tumor gene expression differences statistically evaluated through paired T-tested. Likewise, all patients without a CNV in that same gene had their matched peritoneum to primary tumor gene expression differences paired T-tested together.

To detect the overall affect of CNVs on gene expression we selected all genes within CNVs with expression differences between peritoneal and primary tumors of at least 1.32 fold (log2 0.4 or −0.4). This fold change was used as it is predicted that a single allele amplification or deletion would result in an approximately 1.5 fold change of expression. Data was categorized and averaged by what, if any, copy number variation type it originated from. The same genes were then analyzed without separating the data by copy number variation. That is to say, all patients’ data was averaged for each gene without grouping them by copy number variation type. For comparison, the results from each analyzed group of amplification or deletion was compared to the gene expression differences in patients with no CNV in that gene.

**Figure 2:**
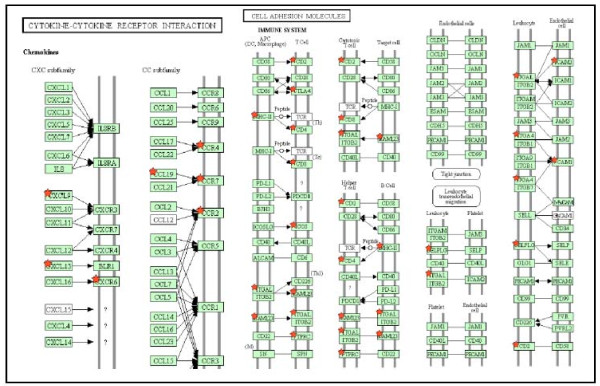
**Pathways enriched in genes differentially expressed between peritoneal metastasis and matched primary tumors.** The significant targeting of these pathways agrees well with results showing copy number variations target the cytokine-cytokine receptor pathway and somatic mutations target the Cell-Adhesion pathways.

Gene expression values for primary and metastatic tumors were normalized in PARTEK using the RMA algorithm. For each gene in a patient's primary/metastatic pair, the primary tumor expression value was subtracted from the metastatic expression value. The paired gene expression differences for all genes in each patient were then subjected to principal components analysis in PARTEK. The top 3 principal components were plotted in Figure 2b.

### Ingenuity pathway analysis

We used Ingenuity Pathway Analysis software (IPA) (Ingenuity Systems, Redwood City, CA) for network analysis of genes that were differentially regulated between primary and metastatic lesions. We constructed networks by overlaying the up and down regulated genes with the gene lists. In the resulting networks genes are represented as nodes, and biological relationships between two nodes as lines. All edges are supported by at least one reference from the literature, textbook, or canonical information stored in the Ingenuity Pathways knowledge database. P-values for enrichment of canonical pathways were generated based on the hypergeometric distribution and calculated with the right-tailed Fisher’s exact for 2 × 2 contingency tables as implemented in Ingenuity.

## Results and discussion

### Direct comparison of primary and metastatic gene expression

**Figure 3:**
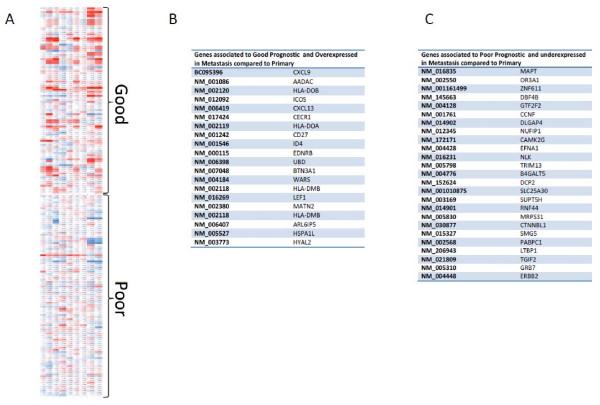
**Peritoneal Metastasis gene expression of TCGA identified prognostic genes. A.** Peritoneal metastasis gene expression was compared to matched tumors for all 9 samples (rows) and gene expression values (columns) were plotted for genes identified by the TCGA to be prognostic of outcome. Peritoneal metastasis has higher expression of good prognostic and lower expression of genes with poor prognosis when compared to the primary metastasis**.** Red represents overexpression, blue represents downregulation. **B.** List of top genes associated to good prognosis and overexpressed in metastasis compared to primary. **C.** List of top genes associated to poor prognosis and downregulated in metastasis compared to primary.

Using the criteria described in the method section a total of 299 probe sets representing 266 annotated genes were differentially expressed in the peritoneal metastases versus the primary tumors. Of these, 202 genes were over-expressed and 64 genes were under-expressed in the peritoneal metastases versus the primary tumors (Figure 1A for a subset of these genes and Additional file 1: Table 1). Some of genes have been previously associated with neoplastic disease in the literature (Figure 1B). Using Ingenuity pathway analysis we retrieved an enrichment in the category “cancer” (25 genes p =1.10-^10^) as well as Reproductive System disease (20 genes, p = 1.10-^10^) Within the cancer category, “ovarian tumor” was the most enriched (p = 1.10-^10^). A network representing all genes implicated in the “cancer” category is represented in Figure 1C and demonstrates the central role of IGF, VEGF, CAV1 and FOS. Functional analysis of the genes using DAVID [12] revealed that over-expressed genes were very significantly enriched in multiple categories. In KEGG pathways, peritoneal over-expressed genes were enriched in ‘Cell Adhesion Molecules’ (12 genes, Benjamini-Hochberg (BH) 1.0x10^-5^), ‘Cytokine-cytokine receptor interaction’ (14 genes, BH score 1.7x10^-3^) (Figure 3) and ‘Jak-STAT signaling pathway’ (8 genes, BH score 0.21) among others. Gene Ontology analysis revealed very significant enrichment for genes with cellular compartment of ‘Plasma Membrane’ (81 genes, BH score 1.9x10^-10^). Likewise, there was very significant enrichment of gene ontology biological processes of ‘Immune Response’ (48 genes, BH score 8.7x10^-24^) and cell activation (29 genes, BH score of 2.5x10^-17^). Interestingly, functional analysis of the 64 down regulated genes did not show significant enrichment in any category.

### Implication of the metastasis on patients’ prognosis

**Figure 4:**
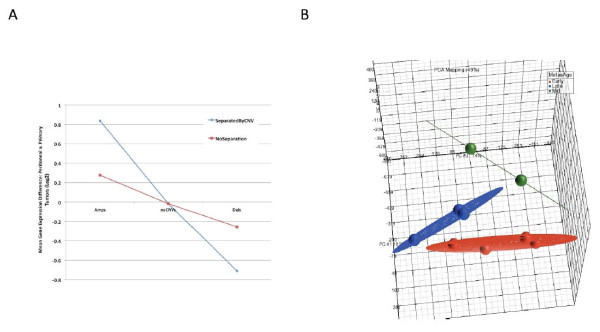
**The effect of underlying copy number variations on gene expression differences between peritoneal and primary tumors. A.** Greater than 2000 genes were used for the analysis. When gene expression data is analyzed by groups of patients containing either amplification (amp), or deletion (del) CNV, the gene expression differences are more significant (SeparatedByCNV). When patients are all analyzed together (NoSeparation), without separating them based on underlying CNVs, the gene expression differences are significantly decreased. As expected, the average gene expression differences between primary and peritoneum in genes outside of CNVs (noCNVs) is near zero. Gene expression data from patients without CNVs do not change. **B.** For each gene in a patient's primary/metastatic pair, the primary tumor expression value was subtracted from the metastatic expression value. The paired gene expression differences for all genes in each patient were then subjected to principal components analysis in Partek.

Recently the TCGA released a large study on ovarian cancer that included gene expression analysis of 489 primary tumors [11]. Analysis of the results yielded gene expression patterns from 193 genes that were prognostic of overall survival. As that comparison was based on primary tumor gene expression differences with relationship to normal tissue, we investigated what the prognostic gene expression pattern was in metastatic lesions versus the primary tumor. For the 193 prognostic genes we recorded gene expression results in the 9 comparisons of peritoneal metastasis versus matched primary tumors. Only genes with detectable expression levels in all samples were used. There was a significant level of good prognosis genes being more highly expressed and poor prognosis genes being lower expressed in the peritoneal metastasis versus matched primary tumors (chi-squared test 30.3, 3 d.f., P < 0.0001) (Figure 4A). This may be indicative of the metastatic lesions remaining closer to normal tissue in their gene expression patterns and is an important consideration when therapies targeting residual disease are considered. Interestingly, Ingenuity pathway analysis of the most overexpressed good prognosis genes (Figure 4B) revealed significant enrichment (more than 5 genes, p < 0.05) of immune networks including “immune cellular movement”, “cell mediated immune response”, and “chemoattraction of lymphocytes”. The genes that were mostly down-regulated in the poor prognosis group (Figure 4C) were significantly enriched for functions such as “cellular movement” (chemoattraction of endothelium, migration of cancer cells), “tumor morphology” (vascularization of tumors) (more than 3 genes, p < 0.05).

### Gene expression analysis guided by CNV analysis

The significant effect of CNVs on level of expressions of genes located within the CNVs or flanking regions is well documented [14,15]. Previous observations on the heterogeneity of ovarian cancer and it’s effect on gene expression [16] and CNV analysis [10], led us to test the effect of CNVs on gene expression in ovarian cancer. Our hypothesis was that underlying heterogeneity in CNVs is likely a source of gene expression heterogeneity. In our previous study we had observed specific pathways consistently affected by CNVs, however, affected genes within those pathways varied among patients. We therefore predicted that accounting for CNVs in the gene expression data might lead to more accurate understanding of which specific pathways were indeed being differentially regulated in subsets of patients. We documented 2945 microarray probe sets, representing 2333 annotated genes, which had both gene expression data for the patients and which were fully contained within a previously identified copy number variation for these samples. We found a very significant effect of underlying copy number variations on gene expression data (Figure 2A). Specifically, when patients were grouped by CNV type for gene expression analysis, the gene expression differences were much more pronounced. As expected, patients with amplifications in a given gene had significantly higher expressions levels for that gene and patients with deletions had significantly lower gene expression levels than did patients with no CNV. Removing the separation of patient data by CNV significantly reduced the observed gene expression difference (Figure 2A).

### Personalized analysis of gene expression

As we discussed above one major drawback of global gene expression analysis is the averaging that may mask major differences between patients. Moreover as we believe that one of the key aspects in ovarian cancer is to pinpoint the differences between the metastatic lesions and the primary tumor we attempted to segregate the metastasis into different groups. One unique aspect of our dataset is our ability to analyze paired primary and metastatic lesions. Principal components analysis of the gene expression differences between pairs of primary and metastatic tumors showed clear separation in three groups. These groups followed a similar separation pattern by metastasis age provided in our analysis of the CNVs [10] demonstrating that some metastasis occur early with low CNVs compare to HapMap while others occur late and have numerous CNVs compare to HapMap and are more similar to the primary tumor (Figure 2B). Specifically, tumors from each metastasis age category clustered closely to each other and separated well from other categories. As expected, tumors from early metastatic events demonstrated the largest numbers of differentially expressed genes when compared to the primary tumors. In the early group 804 probesets were up and 1181 probe-sets were downregulated (> 2 fold change, FDR < 0.2) in metastasis versus primary tumors (Additional file 2: Table 2). In the mid metastasis group the number of genes were greatly reduced with only 89 probe-sets differentially expressed (Additional file 3: Table 3). In this group the patients tend to have metastatic lesions that are closer to the primary tumors as defined by CNV. The increase genomic similarity induces less transcriptomic differences. In the late metastasis no genes were differentially expressed at a statistically significant level.

**Figure 5:**
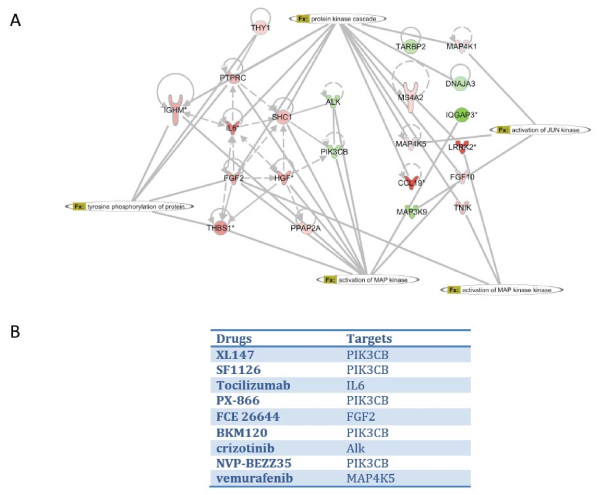
**A. IPA analysis on the gene list obtained in the early metastasis group.** Kinase activation network as predicted by the IPA software. 22 genes implicated in Kinase activation are quite few. **B.** List of predicted inhibitors disrupting disrupt the predicted kinase activation network.

In order to mimic a personalized approach to this issue we performed IPA analysis on the gene list obtained in the early metastasis group. Among the different networks retrieved we focused on the kinase activation network as predicted by the IPA software. Interestingly despite the great number of genes in the global network, the genes implicated in Kinase activation are quite few (22 genes),(Figure 5 A). These genes were mainly upregulated in the early metastasis when compared to their primary tumors. Using the same software we were able to generate a list of drugs able to disrupt the predicted kinase activation (Figure 5 B).

## Conclusions

It is clear from our results that specific pathways in the peritoneum have both been selected for over-expression and amplification (or maintenance of normal expression and avoidance of deletion) with respect to the primary tumor.. While there appears to be some similar specificity in the deletions and under-expression, the enrichment of pathways was less clear for this category. The specific targeting of pathways at both the copy number variation level and the gene expression level in peritoneal metastasis is, to our knowledge, a novel finding in EOC. It would seem that CNVs are both a consequence and cause of gene expression changes. It is likely that the increased gene expression of these pathways is critical to survival in the microenvironment as Wang and colleagues showed that similar pathways are up-regulated in normal peritoneum of patients with ovarian cancer versus those with benign conditions [16]. This requirement would disallow deletion of these genes once the tumor metastasizes to the peritoneum and indeed may be required for the metastasis to occur. On the other hand the primary tumor my delete these genes as they are not required. This has been demonstrated by others in the case of the chemokine gene CCL2 which is deleted in 70% of the tumors investigated and heavily down-regulated in many primary tumors [17]. The study also reported that loss of heterozygosity (LOH) in the CCL2 gene was more frequent in advanced tumors than in earlier stages suggesting it was associated with progression and not initiation of the cancer. Our combined gene expression and CNV analysis of CCL2 suggests that gene expression and copy number are maintained in the peritoneal metastasis while frequently being deleted in the matched primary tumors. These differences in CCL2 copy between the primary tumor and metastatic lesions should help guide the future consideration of chemotherapeutic targets to avoid targeting primary tumor specific alterations only. It is interesting to note that ‘Immune Response’ genes, which includes the cytokines, was heavily enriched in over-expressed genes in the peritoneum metastasis. This is unlikely to be solely due to immune cell infiltrate in the samples collected as CNV data showed a related enrichment for many of the same genes. The enrichment for immune response genes was expansive regardless whether or not CNV data was taken into account. Indeed, even in 20 ‘immune response’ related genes that were deleted in the peritoneum, gene expression was elevated with respect to the primary tumor. This indicates that there are likely transcriptional and not just genomic causes for increases gene expression in this biological process.

As predicted, we did find a significant correlation between the type of CNV and the effect on gene expression. This correlation is important for future analysis as typical gene expression studies seek differential expression of at least 3 fold. Given our findings, it is likely that there are many genes whose gene expression is affected by CNVs but for which the change (approximately 1.5 fold with gain/loss of single allele) is too small to detect. This may be the underlying cause of heterogeneity in studies of primary and metastatic lesions in EOC. When patient samples are compared simply based on gene expression, the heterogeneity may result in averaging to insignificance for a given gene. We have shown that, by analyzing gene-expression data in light of CNV data, we recover more differentially expressed genes reported by previous studies. This is especially true in the case of metastases that occur earlier and remain less affected by CNVs than do their later counterparts. This approach will be critical in truly identifying pathways rather than specific genes that are targeted in ovarian cancer metastasis.

Along these lines, our analysis of the early metastasis group revealed a network of kinases with existing chemotherapeutic agents. This method of individually studying patients’ metastases should lead to a more effective, personalized medicine. The identification of differences between metastatic and primary tumors leads to two concepts: (i) differences between the metastasis and primary tumors will require therapies targeting their specific genetic alterations [18,19]; (ii) It becomes critical to focus significant research on metastatic disease, and several important aspects remain to be addressed such as: levels of heterogeneity of metastatic lesions within the same patient, and the biology of recurrent or residual disease compared to the primary and metastatic disease.

Answering these primordial questions in ovarian cancer will help us design personalized approaches to the disease using a more targeted and comprehensive approach than the analysis of the primary tumor alone.

## Competing interests

The Authors declare no competing interests.

## Authors’ contribution

JM, AR, DQ designed the study, ran analysis and wrote the manuscript; GF, AR, DQ have sampled the patients, AM contributed to coordination with pathology department, processing of the tumor samples, drafted the manuscript and retrieved clinical data, EJ retrieved clinical data, RH, JPT, CR performed the CNV and transcriptomic arrays as well as controls, EM performed all histology controls. All authors read and approved the final manuscript.

## Supplementary Material

Additional file 1 **Table 1.** Gene expression differences between primary and metastatic lesions ( 1.5 fold change and paired T-test p-value of less than 0.01). Click here for file

Additional file 2 **Table 2.** Gene expression differences between primary and metastatic lesions in the early group (2 fold change and paired T-test p-value of less than 0.01). Click here for file

Additional file 3 **Table 3.** Gene expression differences between primary and metastatic lesions in the mid group (2 fold change and paired T-test p-value of less than 0.01). Click here for file
